# Tetramethylpyrazine ameliorates acute lung injury by regulating the Rac1/LIMK1 signaling pathway

**DOI:** 10.3389/fphar.2022.1005014

**Published:** 2023-01-06

**Authors:** Simin Min, Weiting Tao, Dushan Ding, Xiaonan Zhang, Shidi Zhao, Yong Zhang, Xiaojie Liu, Kefei Gao, Saisai Liu, Li Li, Min Hou, Yan Li

**Affiliations:** ^1^ School of medicine and health engineering, Changzhou university, Changzhou, Jiangsu, China; ^2^ Department of Pathophysiology, Bengbu Medical College, Bengbu, Anhui, China; ^3^ Department of Respiratory, The First Affiliated Hospital of Bengbu Medical College, Bengbu, Anhui, China; ^4^ Department of Pharmaceutical Engineering, Bengbu Medical College, Bengbu, Anhui, China

**Keywords:** tetramethylpyrazine, tight junction, pulmonary capillary, endothelial cells, acute lung injury, LPS

## Abstract

Acute lung injury (ALI) is a respiratory disorder characterized by severe inflammation of the alveoli and lung parenchyma. Tetramethylpyrazine (TMP), the main active compound in Ligusticum chuanxiong Hort (LC), can protect against lipopolysaccharide (LPS)-induced ALI. Our study aimed to investigate how TMP protects the endothelial cell barrier in pulmonary capillaries. We administered TMP intraperitoneally at different doses and found that acute lung injury in mice was improved, but not in a dose-dependent manner. TMP toxicity was tested *in vitro*. We observed that LPS-induced cytoskeletal remodeling was inhibited by TMP. Murine ALI was induced as follows: For the 1st hit, LPS (2 mg/kg) was injected intraperitoneally; after 16 h, for the 2nd hit, LPS (4 mg/kg) was instilled intratracheally. The mice in treatment groups had TMP or dexamethasone administered intraperitoneally 30 min prior to the 1st hit and 30 min past the 2nd hit. Mice were euthanized 24 h after the last injecting. We measured protein and mRNA levels using enzyme-linked immunosorbent assay (ELISA) and reverse transcriptase real-time PCR (RT-qPCR), respectively. The ultrastructural analysis was performed with transmission electron microscopy (TEM) and the cytoskeleton was observed by immunofluorescence. Immunohistochemistry and Western blotting were used to detect protein expression in the Rac1/LIMK1/ZO-1/occludin signal pathway. The results showed that TMP treatment decreased inflammatory cell infiltration and alleviated LPS-induced damage in lung tissue. Also, TMP significantly inhibited the Rac1/LIMK1/ZO-1/occludin signaling pathway. Our findings show that using TMP during sepsis can protect the pulmonary microvascular endothelial cell barrier and suppress inflammation. Therefore, TMP may have a promising therapeutic role in preventing acute lung injury from sepsis.

## Introduction

Acute lung injury (ALI) is the pulmonary manifestation of a systemic inflammatory response syndrome that can originate from direct or indirect factors such as corrosive chemicals, toxins, or inflammatory mediators (Jiang et al., 2016; Belvitch et al., 2020). It has a high mortality rate of over 40% (Singer et al., 2016). A key pathologic process of ALI is pulmonary vascular endothelial barrier dysfunction leading to microvascular leakage (Aird, 2003). The Rho family of small GTPases, including Rho, Rac, and Cdc42 are important regulators of cytoskeleton dynamics. Rho/Rac activation leads to cytoskeletal rearrangement, resulting in the contraction of endothelial cells and widening of intercellular spaces that ultimately increase vascular permeability (Tzima et al., 2002). Endothelial cells form a semi-permeable barrier that controls the movement of cells, fluids and solutes across the vessel walls (Sheikpranbabu et al., 2009). Although the edema and inflammation caused by pulmonary endothelial cell injury are critical in the pathogenesis of ALI, the complete mechanism is still unclear and requires further study.

Lipopolysaccharides (LPS), also known as endotoxins, can induce endothelial cell barrier damage both *in vivo* and *in vitro* (Catravas et al., 2010; Li Y. et al., 2021). LPS-induced ALI is characterized by neutrophils sequestration into the lung, overproduction of inflammatory mediators, increased vascular permeability, and distortion of the pulmonary epithelium barrier (de Souza Xavier Costa et al., 2017; Huang et al., 2017).

Rac1 and RhoA are Rho GTPase subfamily members that play key roles in cytoskeletal structure (Zhang J.-G. et al., 2020). Rac1 Activation stimulates actin polymerization into filaments. Rac1 has been found to inhibit RhoA activity *via* a variety of mechanisms (Guilluy et al., 2011; Hetmanski et al., 2018). In addition, Rho kinase (ROCK) can suppress Rac1-GTP (Sanz-Moreno et al., 2008). Therefore, a treatment that targets the Rac1 signaling pathway and inhibits cytoskeleton remodeling may improve ALI prognosis.

Tetramethylpyrazine (ligustrazine, TMP) is an important constituent of the Chinese herb, *Chuanxiong Rhizoma* (CR), which is widely used in China to treat cardiovascular diseases (CVD) (Liu et al., 2010; Fan et al., 2011; Yu et al., 2013). However, research on the application and mechanism of TMP on ALI is still in its infant stage. Our previous study confirmed that TMP can prevent LPS-induced ALI by the Rho/ROCK pathway (Zhao et al., 2015). Recent studies have shown that TMP therapy for ALI is at least partially regulated by Rac1 expression in endothelium, but its role in LPS-induced endothelial barrier disruption is uncertain. The Rac1/LIMK1 signaling pathway activity is prominent in the cytoskeletal structure, and any abnormal activation can rearrange the cytoskeleton (Li et al., 2020; Wufuer et al., 2021). Tight junctions (TJs) involve close apposition of transmembrane proteins between cells. Zonal occludens-1 (ZO-1) links TJ molecules to the actin cytoskeleton (Chen et al., 2015; Feng et al., 2018). Mainly tight junction destruction and endothelial damage cause LPS-induced distortion of the alveolar-capillary barrier. We will discuss the therapeutic effects and mechanisms of TMP on mice with acute lung injury. Possibly, TMP can inhibit microvascular endothelial cell permeability and reduce pulmonary inflammation and lung injury by inhibiting the Rac1/LIMK1/ZO-1/occludin pathway.

## Materials and methods

### Materials

Tetramethylpyrazine was obtained from the National Institute for the Control of Pharmaceutical and Biological Products (Beijing, China). LPS and fluorescein isothiocyanate (FITC)-dextran were purchased from Sigma-Aldrich (Saint Louis, United States). Dexamethasone was purchased from Solarbio (Beijing, China). Endothelial cell medium (ECM) was purchased from ScienCell (Carlsbad, United States). Umbilical cords were obtained from the First Affiliated Hospital of Bengbu Medical College (Anhui, China). M199 medium, protease inhibitors, RIPA cell lysis kit, DAPI staining solution, and horseradish peroxidase (HRP) -labeled anti-rabbit or anti-mouse antibodies were purchased from Biosharp (Hefei, China). The 4% paraformaldehyde was acquired from Beyotime (Shanghai, China). The VWF antibody, ELISA kits, and the anti-fluorescence quencher were purchased from Wuhan Boster Biological Technology (Wuhan, China). The anti-p-Rac1, anti-Rac1, anti-LIMK1, anti-ZO-1 antibodies and anti-Occludin were purchased from Abcam Company (Toronto, Canada), and anti-p-LIMK1 from Affinity (United States). Anti-GAPDH was purchased from ABclonal Science, Inc. (Wuhan, China). The qPCR reagents were purchased from Vazyme Biotech (Nanjing, China).

### Cell cultures and reagents

Endothelial cells were from the umbilical cord of healthy mothers who had normal term deliveries at the First Affiliated Hospital of Bengbu Medical College. Under aseptic conditions, the umbilical cord samples were first washed with PBS and then disinfected on the surface with 75% alcohol. The vein in the umbilical cord was flushed with PBS. The cords were digested by .1% type I collagenase at 37°C, and the digestive solution was collected in 50 ml centrifuge tubes. The rinse solution was also collected in the centrifuge tube, and the supernatant was discarded after centrifuging at 1,000 rpm for 10 min. In a new culture bottle, the cells were transferred to a CO_2_ incubator (37°C, 5% CO_2_) after being redissolved in endothelial cell medium. After 36 h, the media was changed, and cells were digested with pancreatin (Biosharp, China) and passaged. The human umbilical vein endothelial cells (HUVECs) from passages 2 to 4 were used in subsequent experiments.

### A “double-hit” LPS mouse model

Approximately 20 g average weight C57/BL6 male mice (around 7 weeks old) were purchased from the Hangzhou ZiYuan Technology Co., Ltd., China (Certification number: SCXK 2017-0003). A specific pathogen-free (SPF) environment was maintained with free access to standard rodent chow during 12-h day and night cycles (22°C ± 2°C, 50% ± 5% humidity). Previously, we reported that “double-hit” induced lung injury models were more similar to clinical acute lung injury (Zhao et al., 2015). To explore the effects of TMP in mice, the ALI was induced by a “double-hit” of LPS. LPS (2 mg/kg) was injected intraperitoneally into the mice. After 16 h, intratracheal instillation with LPS (4 mg/kg) was done. Different doses of TMP were intraperitoneally injected 30 min before LPS injection and 30 min after tracheal infusion. The doses used were: 40 mg/kg (low dose, TMP-L), 80 mg/kg (middle dose, TMP-M), and 120 mg/kg (high dose, TMP-H). The mice were randomly allocated to the following six experimental groups (n = 10): 1) PBS control 2) LPS + PBS 3) LPS + TMP-L 4) LPS + TMP-M 5) LPS + TMP-H, and 6) LPS + DEX. Each group had 7 to 10 mice. We collected lung tissues and bronchoalveolar lavage fluid (BALF) 24 h after intratracheal LPS instillation. Samples were kept in freezer at −80°C until further analysis.

### Measurement for lung inflammation

Lung inflammation in each experimental group was measured using BALF cell count and ELISA of cytokines and chemokines in lung tissues. By sharp dissection after euthanasia, the mice chests and tracheas were exposed. Using a 1 ml syringe, the trachea was cannulated and the lung lavage performed 4 times with 0.75 ml cold PBS. After centrifuging the BALF sample for 5 min at ×600 g, 100 μL cold PBS was used to resuspend the cell pellets, mixed well, then 100 μL was placed into another centrifuge tube and 450 μL of leukocyte dilution was added. Cell counter plates were used to count white blood cells in BALF. Lung tissue was collected into a centrifuge tube, placed in tissue lysate, and homogenized on ice using a tissue homogenizer. The serum was collected to analyze TNF-α, IL-6, and IL-1β by ELISA.

### Assessment of lung microvascular leak

The ratio of wet-to-dry lung weight and protein concentration in BALF were used to assess lung microvascular leakage. The wet weight of left lung tissue was measured, then the dry weight of the tissue was measured after 48 h of being placed in an oven at 70°C. The concentration of protein in BALF was measured with the BCA Protein Assay Kit.

### Histological analysis and lung injury scoring

Lung tissues of mice were harvested 24 h after intratracheal LPS instillation and fixed with 4% paraformaldehyde. After fixation, all tissues were processed in an automated tissue processor for dehydration and paraffin embedding. They were then sectioned into 5 μm-thick slides. A photomicroscope (Zeiss inverted fluorescence microscope) was used to examine the slides stained with hematoxylin and eosin (H&E). A pathologist blinded to the groups conducted histological analysis and lung injury scoring.

### Validation test for quantitative real-time PCR (RT-qPCR)

The total RNA was extracted using TRIzol reagent according to the manufacturer’s instructions and RNA reverse transcription was performed using a reverse transcription kit. The PCR reaction system was created using 20 µL of a solution containing 10 µL of ×2 AceQ Universal SYBR qPCR Master Mix, 2 µL of diluted cDNA, .4 µL each of the forward and reverse primers, and 7.2 µL of ddH_2_O. The PCR reaction was performed under the following conditions: step 1: 95°C for 10 min; step 2: 95°C for 10 s and 57°C for 30 s; and step 3: 95°C for 15 s 60°C for 60 s and 95°C for 15 s (repeat step 2 for 40 cycles). PCR amplification of TNF-α, IL-1β, IL-6, and GADPH was performed by Rcvhe-LightCycler480. Primers were synthesized by Sangon (Shanghai, China).

### Western blotting

We extracted protein from lung tissue using RIPA lysis buffer. Protein lysates (20 μg) from each sample were mixed in SDS-loading buffers and boiled at 100°C for 5 min. The mixture was run on a 10%–12.5% SDS-PAGE gel, transferred into PVDF membrane, which was then blocked with 5% BSA for 2 h in room temperature. A combination of antibodies against LIMK1 (1:1500), p-LIMK1 (1:1000), Rac1 (1:1000), p-Rac1 (1:750), ZO-1 (1:1000), occludin (1:1000) and GAPDH (1:10000) was then incubated overnight at 4°C.

### Immunohistochemistry (IHC)

The lung tissues of mice were incubated in 4% paraformaldehyde for fixation and paraffin embedding. After dewaxing in xylene, paraffin-embedded sections were dehydrated in graded ethanol. Incubation for 30 min with 0.3% H_2_O_2_ in PBS was followed by blocking with PBS containing 1% BSA for 1 h. Proteins of interest were recognized by incubation with anti-MPO (Cell Signaling, 1:1000) at 4°C overnight. After washing with TBST, sections were incubated at room temperature for 30 min with SignalStain Boost IHC Detection Reagent (CST, United States). SignalStain DAB (Cell Signaling Technology) was then applied to the sections to reveal the intensity of the staining. Sections were captured using an Olympus BX-51 microscope (Olympus, Japan).

### Statistical analysis

Data were presented as mean ± SD. All statistical analyses were conducted using GraphPad Prism software 9.0 (GraphPad Software, Inc., United States). A single-factor analysis of variance was used to compare the groups. Significant results were defined as *p*-values below 0.05.

## Results

### LPS causes lung injury in mice

**FIGURE 1:**
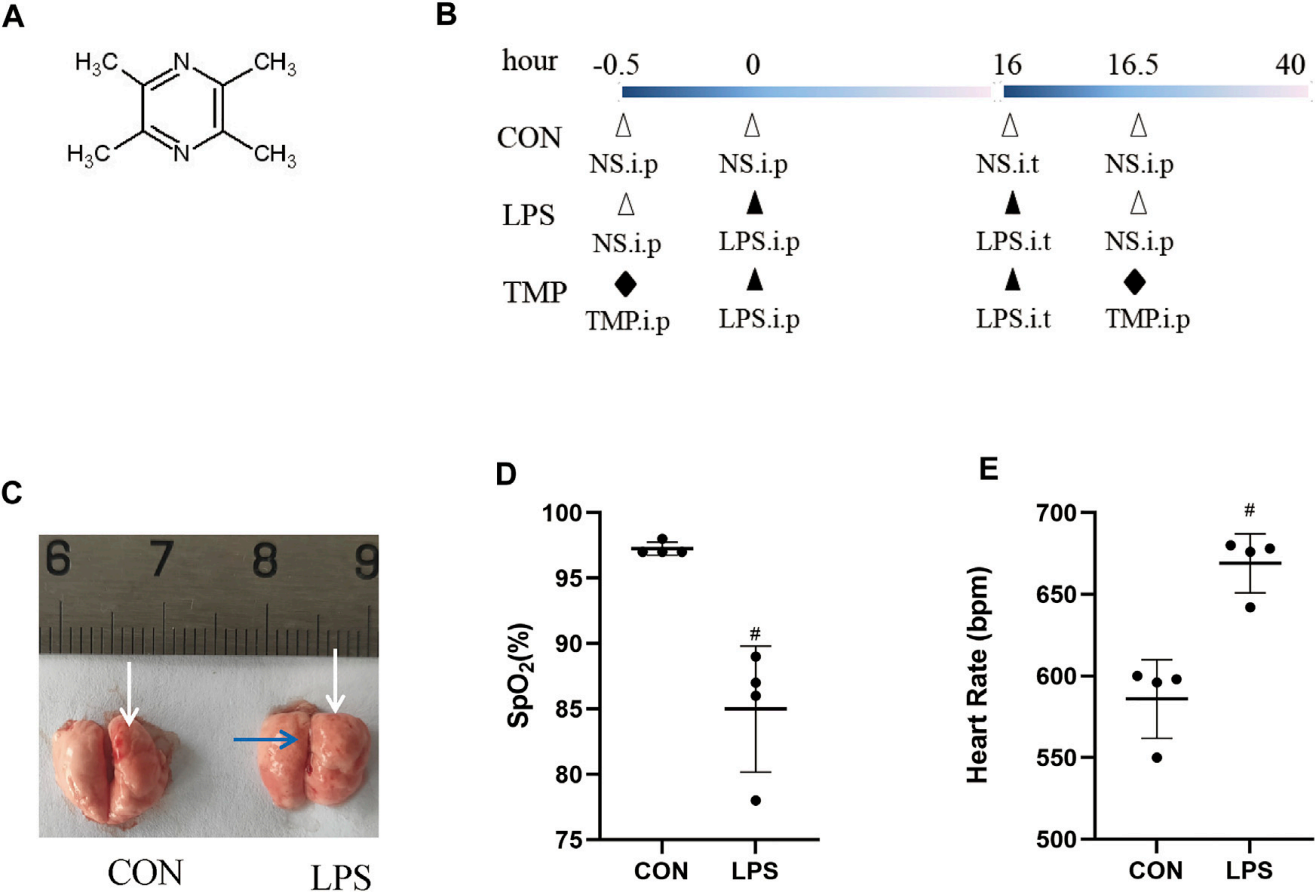
Lung injury induction by LPS. **(A)** The structural formula of TMP. **(B)** TMP treatment protocol in C57BL/6 mice with LPS-induced ALI. **(C)** Lung photographs (in control and LPS group; white arrows indicate the pulmonary septa, the blue arrow indicates pulmonary edema). **(D)** Peripheral blood oxygen saturation in both groups. **(E)** Heart rate of mice in both groups. ^#^ *p* < 0.05 versus CON group, *n* = 4 per group.

TMP plays an important role in inflammation and microcirculation improvement. We evaluated the protective action of TMP on LPS-induced murine ALI (Figure 1B). The mice had obvious lung edema evidenced by the reduced space between the left and right lungs and shiny lung surface (Figure 1C). The diagnostic criteria for acute lung injury include arterial blood gas analysis that assesses the degree of hypoxemia using the ratio of arterial partial pressure of oxygen (PaO_2_) to inhaled oxygen concentration (FiO_2_) (PaO_2_/FiO_2_). Because the ALI mouse model wasn't supported by a ventilator, we chose to use a significant and sustained decrease in peripheral blood oxygen saturation (SpO_2_) instead of the Berlin standard PaO_2_/FiO_2_ ratio. In this study, the SpO_2_ of the mice decreased significantly (>10%) within 24 h, and their heart rate increased significantly (Figures 1D,E).

### TMP protects lungs in mice with ALI

**FIGURE 2:**
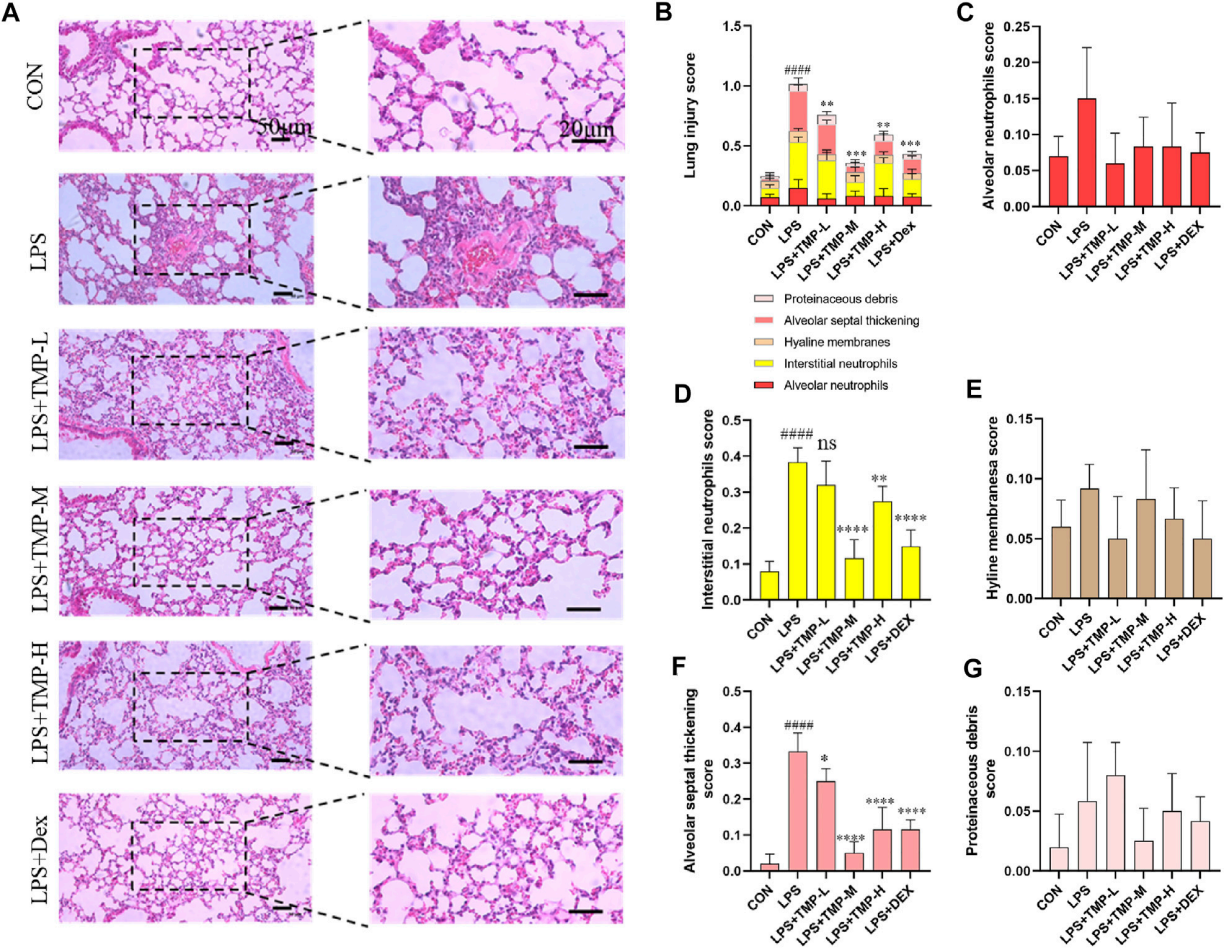
TMP improved lung injury. **(A)** Images of H&E staining in all groups. **(B)** Total lung injury score based on the H&E images. **(C)** Alveolar neutrophils score. **(D)** Interstitial neutrophils score. **(E)** Hyaline membranes score. **(F)** Alveolar septal thickening score. **(G)** Proteinaceous debris score. TMP-L = 40 mg/kg, TMP-M = 80 mg/kg, TMP-H = 120 mg/kg, Dex = 3 mg/kg ^####^ *p* < 0.0001 versus CON group, ^*^ *p* < 0.05 versus LPS group, ^**^ *p* < 0.01 versus LPS group, ^***^ *p* < 0.001 versus LPS group, ^****^ *p* < 0.0001 versus LPS group, *n* = 6 per group.

Using histopathologic improvements, we compared six groups including three TMP dose groups and a positive control group (dexamethasone, Dex). The histopathologic analysis of lungs with ALI showed that TMP treatment effectively decreased lung inflammation and injury (Figure 2). Variable doses of TMP (TMP-L = 40 mg/kg, TMP-M = 80 mg/kg, TMP-H = 120 mg/kg) could inhibit LPS-induced severe inflammatory cell infiltration (Figure 2A). We quantitatively assessed the LPS-induced ALI using the following five histologic features of mice lungs: alveolar neutrophils, interstitial neutrophils, hyaline membranes, proteinaceous debris, and alveolar septal thickening (Figure 2B) (Davies et al., 2015). No change was observed in alveolar space neutrophils (Figure 2C). Interstitial neutrophil infiltration was apparent in the lung tissue with LPS-induced murine ALI. In the TMP and Dex treatment groups, there was an obvious morphologic improvement in the lung after treatment (Figure 2D). Furthermore, hyaline membranes formed and proteinaceous debris was present, as well as thickening of alveolar septum, but hyline membranesa score and proteinaceous debris remained insignificant between the groups (Figures 2E,G). For mice in the treatment groups, alveolar septal thickening showed significant improvement, particularly in the medium-dose TMP group (Figure 2F).

### TMP alleviates LPS-Induced lung inflammation in mice

**FIGURE 3:**
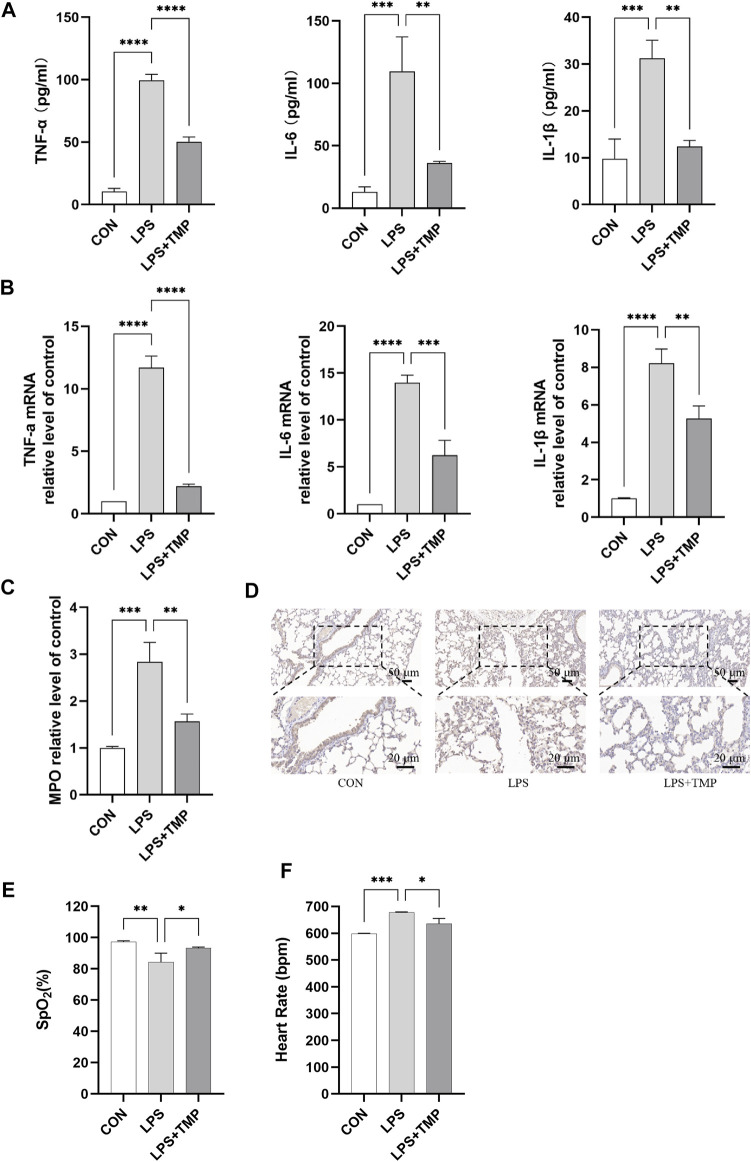
Mice’s lung function and inflammation were affected by TMP. **(A)** Serum levels of inflammatory factors in mice. **(B)** Inflammatory cytokines mRNA levels of mice lung tissue. **(C,D)** Detection of myeloperoxidase activity by ELISA and immunohistochemistry. **(E)** Peripheral oxygen saturation (SpO_2_). **(F)** Heart rate. ^**^ *p* < 0.01, ^***^ *p* < 0.001, ^****^ *p* < 0.00001, *n* = 3 per group.

H & E staining showed that medium-dose TMP had the most effect on lung injury in mice. So, we further examined the effect of medium-dose TMP on inflammatory factors in mice. ELISA results indicated that LPS significantly increased the expression and secretion of proinflammatory cytokines in the serum of ALI mice, which significantly decreased in mice pretreated with TMP (Figure 3A). We found that TMP could significantly inhibit TNF-α, IL-6, and IL-1β expression levels using RT-qPCR in lung tissues of ALI mice (Figure 3B). ELISA and immunohistochemistry results also showed that TMP inhibited LPS-induced elevation of myeloperoxidase (MPO) expression (Figures 3C,D). The SpO_2_ was significantly increased (Figure 3E) and the heart rate significantly decreased (Figure 3F) in the group treated with TMP compared to the control group.

### TMP inhibits LPS-induced cytoskeletal remodeling and apoptosis *in vivo*

**FIGURE 4:**
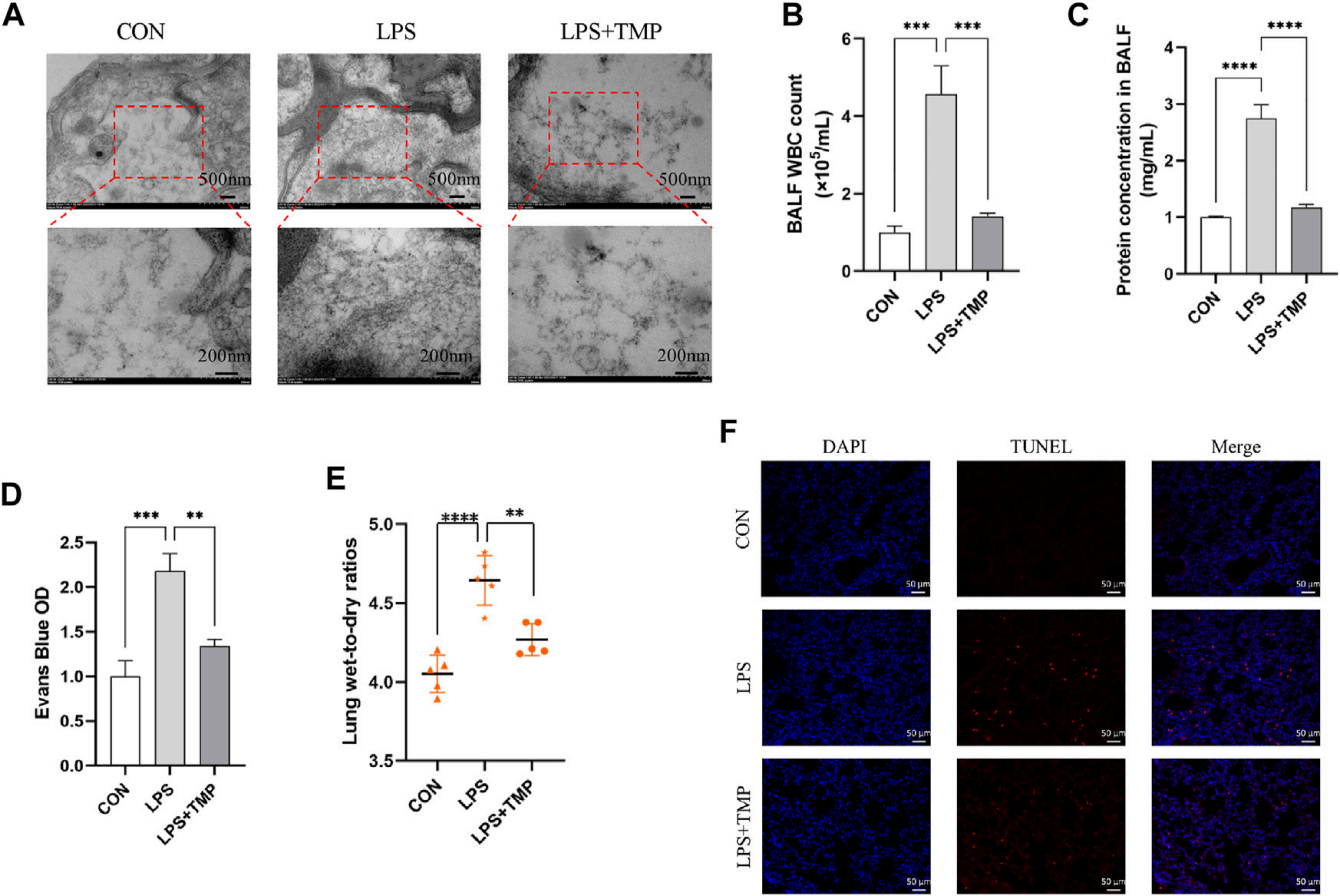
TMP inhibited LPS-induced cytoskeletal remodeling and apoptosis *in vivo*. **(A)** The cytoskeleton was observed by TEM. **(B–C)** The WBC and total protein contents in BALF. **(D)** Evans Blue OD. **(E)** Lung wet-to-dry ratios. **(F)** LPS-induced cell apoptosis of all groups (scale bar = 50 μm). ^**^ *p* < 0.01, ^***^ *p* < 0.001, ^****^ *p* < 0.00001, *n* = 5 per group.

After LPS administration, the cytoskeleton was remodeled, stress fibers became thicker, shorter, and disordered, endothelial cells contracted, cell permeability increased, microvascular leakage occurred, and TEM showed an increase and thickening of actin microfilaments. TMP treatment inhibited LPS-induced endothelial cytoskeletal remodeling (Figure 4A). Increases in BALF cells number, protein concentration and wet-to-dry lung weight ratio are markers of enhanced endothelial permeability. The BALF obtained 24 h after intratracheal instillation with LPS showed significantly increased cell count and protein concentration levels. These effects were mitigated by intraperitoneal administration of TMP (Figures 4B,D). Moreover, the mice had obvious pulmonary interstitial edema. TMP treatment significantly attenuated the increase in wet-to-dry weight ratio (Figure 4E). LPS-induced apoptosis was significantly aggravated compared to controls. TMP treatment could effectively inhibit apoptosis (Figure 4F).

### TMP reduces LPS-induced HUVEC hyperpermeability and death *in vitro*

**FIGURE 5:**
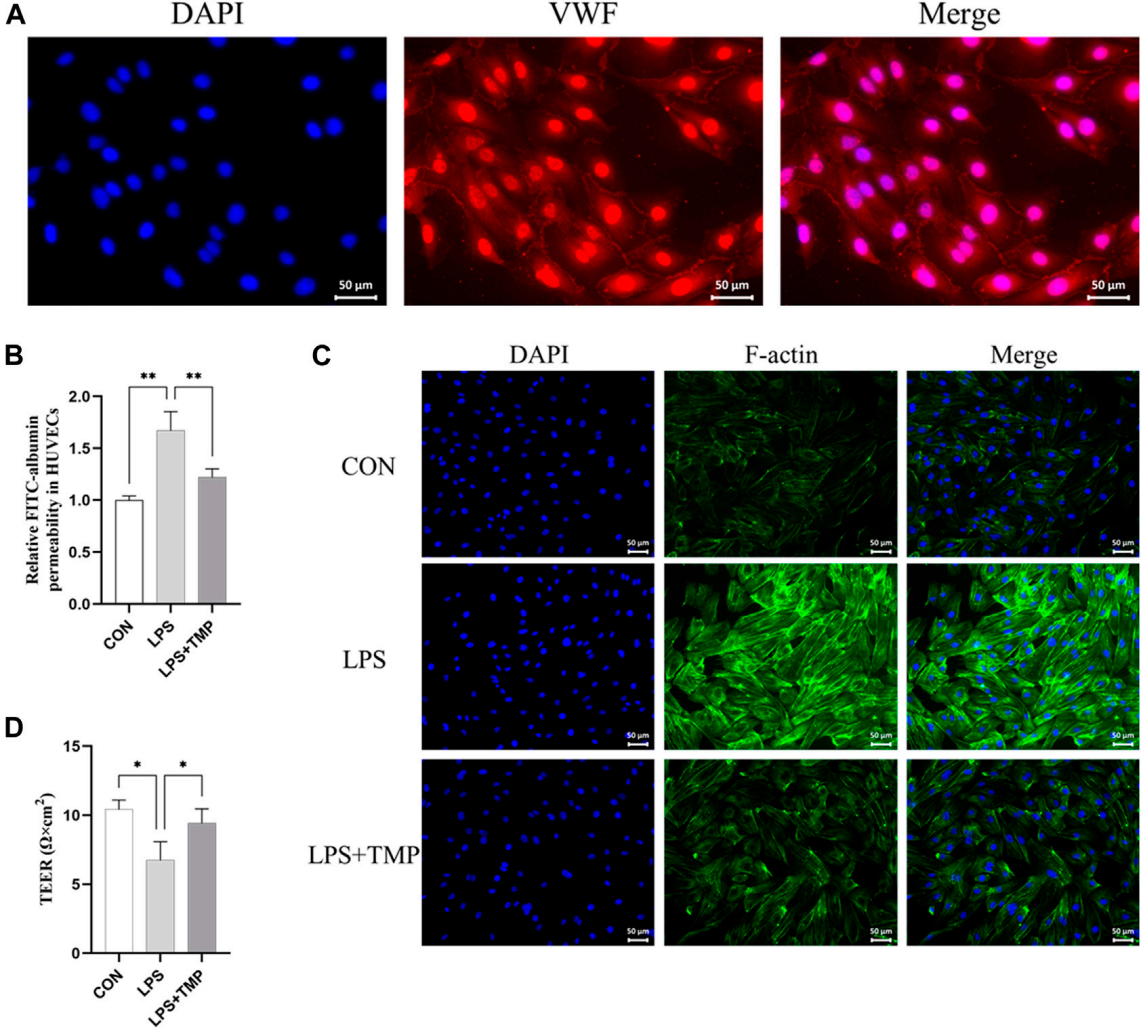
Effects of TMP on the cytoskeleton *in vitro*. **(A)** VWF staining was used to identify endothelial cells (scale bar = 50 μm). **(B)** Transwell chamber FITC albumin was used to determine the effect of TMP (15 ng/ml) on HUVEC after 12 h of LPS (200 ng/ml) exposure. **(C)** Immunofluorescence assay for the cytoskeleton (scale bar = 50 μm). **(D)** Transendothelial electrical resistance (TEER) in TMP-treated, untreated, and control HUVECs. ^*^ *p* < 0.05, ^**^ *p* < 0.01, *n* = 3 per group.

Our experiment was designed to test the effect of TMP on LPS-induced cytoskeletal remodeling and death of HUVECs. We first identified HUVECs (Figure 5A). The viability of the cells was above 85% for TMP concentration below 15 ng/ml (Supplementary Figure S1). Changes in the endothelial cytoskeleton could affect endothelial permeability. As expected, TMP ameliorated FITC-albumin permeability in HUVECs (Figure 5B) and LPS-induced cytoskeletal remodeling (Figure 5C). HUVEC treated with TMP had significantly higher transendothelial electrical resistance values (TEER) than untreated HUVEC. This indicates that TMP treatment can reduce LPS-induced cell permeability (Figure 5D). The results suggest that TMP reduces LPS-induced HUVEC permeability and cytoskeletal remodeling.

### TMP attenuates murine ALI through the Rac1/LIMK1 pathway

**FIGURE 6:**
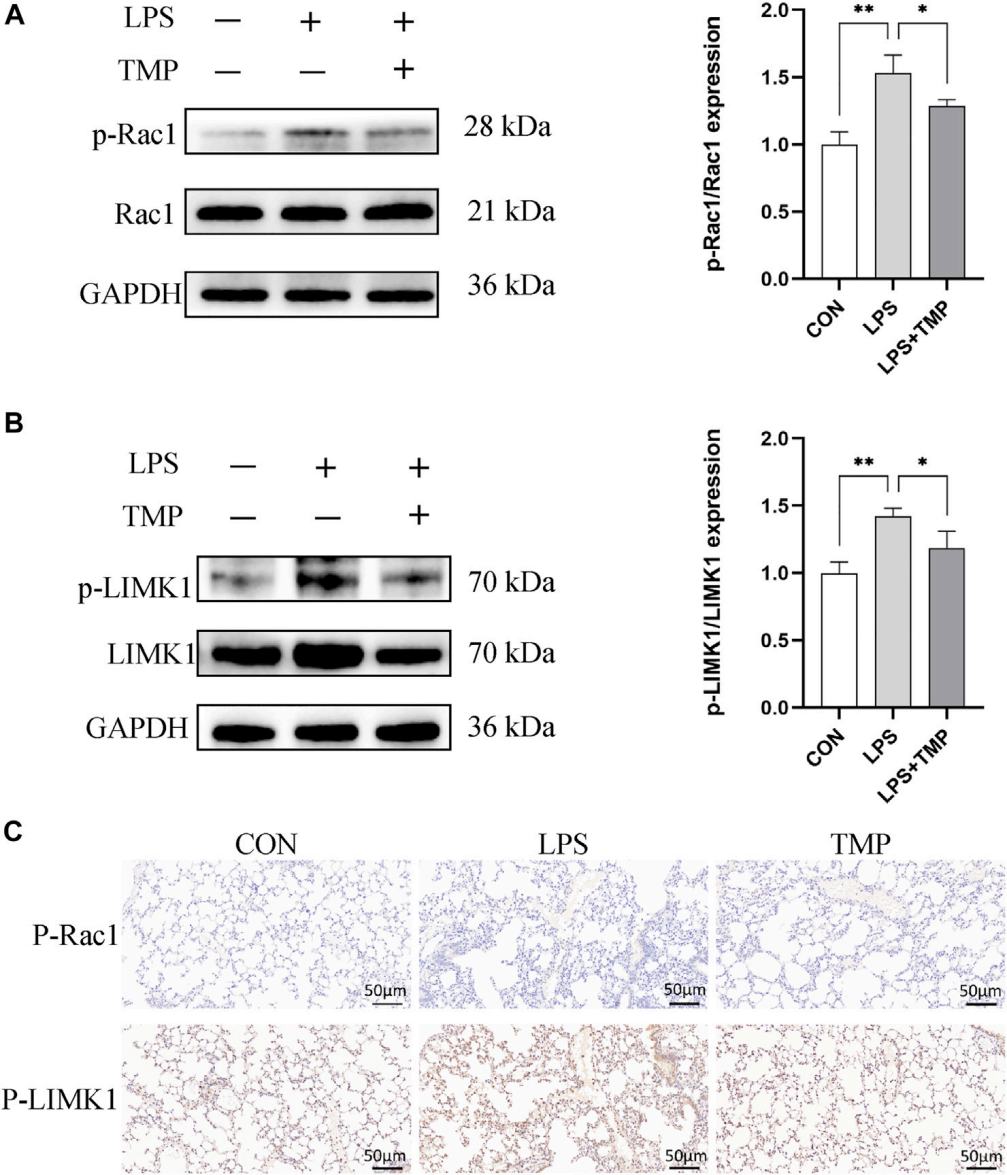
TMP protects pulmonary microvascular endothelial cells in murine ALI *via* Rac1/LIMK1 pathway. **(A)** The inhibitory activity of TMP on Rac1 phosphorylation (p-Rac1) and **(B)** LIMK1 phosphorylation (p-LIMK1). **(C)** IHC staining of the p-Rac1 and p-LIMK1 in mice lung tissue. ^*^ *p* < 0.05, ^**^ *p* < 0.01, *n* = 3 per group.

The binding mode of TMP and Rac1 was predicted by the molecular docking method (Supplementary Figure S2). The Rac1/LIMK1 pathway plays a key role in regulating cytoskeleton and endothelial permeability. We investigated the Rac1/LIMK1 pathway in mice with LPS-induced ALI with or without TMP pretreatment to determine how TMP induces its anti-inflammatory effect. In this study, TMP ameliorates LPS-induced phosphorylation of Rac1 and LIMK1. Previous studies have shown that an increase of p-LIMK1 and p-Rac1 results in cytoskeletal remodeling and endothelial hyperpermeability. Our results show that LPS increased p-LIMK1 and p-Rac1 and pretreatment with TMP significantly inhibited this increase (Figures 6A,B). Immunohistochemistry revealed the same trends (Figure 6C).

### TMP ameliorates LPS-induced tight junction impairment

**FIGURE 7:**
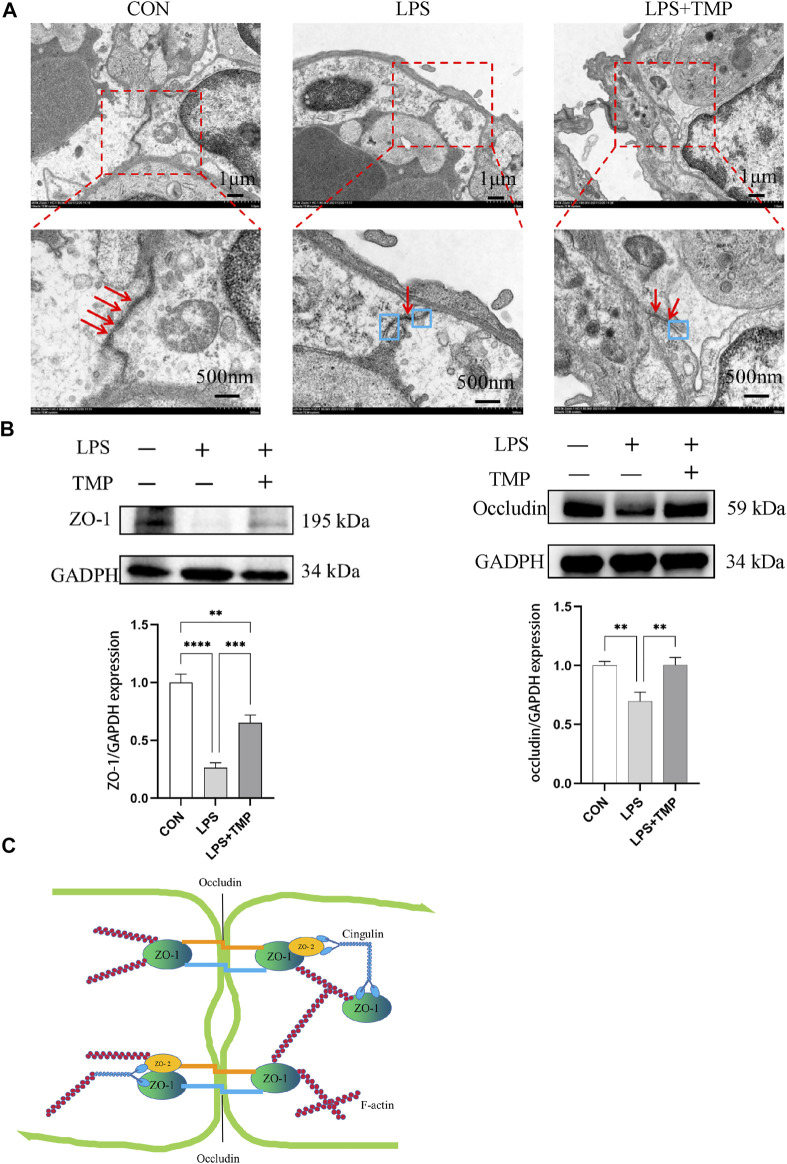
Impact of TMP on tight junction connections. **(A)** TEM observation of tight junction connections (red arrows represent tight connections, blue boxes represent separation of tight junction connections). **(B)** ZO-1 and Occludin protein levels were evaluated by Western blotting. **(C)** F-actin activation decreased ZO-1 and Occludin. ^**^ *p* < 0.01, ^***^ *p* < 0.001, ^****^ *p* < 0.00001, *n* = 3 per group.

Tight junctions are closely related to cellular permeability, which increases when tight junctions are damaged. Pulmonary capillary endothelial cells were observed by electron microscopy. In normal mice, the pulmonary capillary endothelial cells were closely connected with continuous strips, and no gaps were observed between the cells. LPS damaged the continuous tight junctions of endothelial cells with some showing obvious cracks. TMP treatment ameliorated this (Figure 7A). The treatment of LPS could decreased expression levels of tight junction proteins including ZO-1 and occludin, but TMP pretreatment inhibited this reduction (Figure 7B). LPS directly or indirectly (*via* cytokines) stimulates Rac1 and then induces LIMK1 activation, which further activates F-actin and increases paracellular permeability (Figure 7C).

## Discussion

Tetramethylpyrazine (TMP) is a bioactive ingredient of the medicinal plant *Chuanxiong Rhizoma* (CR) (Lu et al., 2014). Although TMP has therapeutic effects on different ALI models (Hu et al., 2020; Li J. et al., 2021), its precise molecular mechanism of action remains unclear. Our study confirmed that pretreatment with TMP improved the oxygen saturation of mice with LPS-induced ALI, reduced pathologic lung damage, alleviated lung inflammation, reduced myeloperoxidase (MPO) activity, reduced BALF protein and neutrophil concentration *in vivo*, and blocked cytoskeletal reorganization. These results provide convincing evidence for using TMP to treat LPS-induced ALI.

**FIGURE 8:**
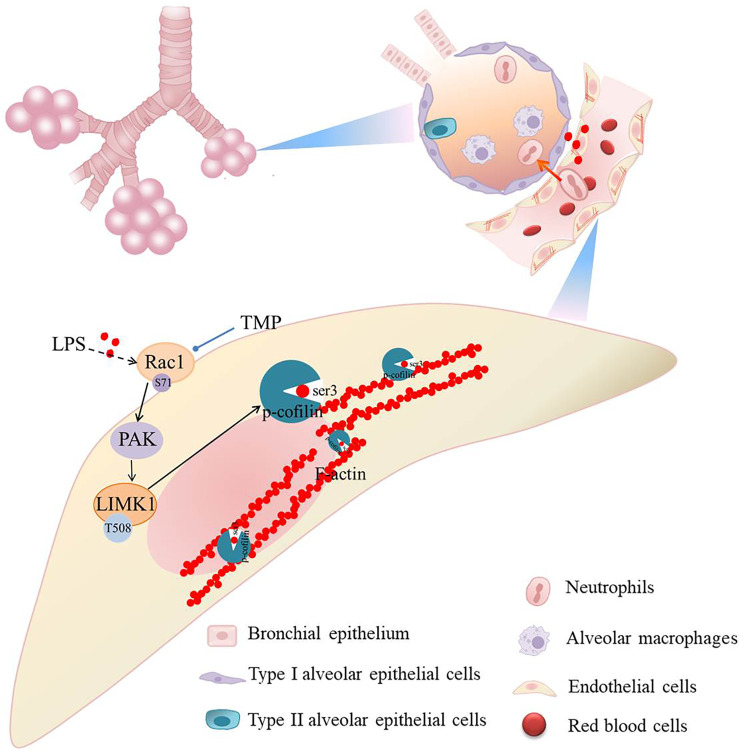
Schematic diagram of TMP on Rac1/LIMK1 signaling pathway.

We have virtually screened the binding sites of TMP and Rac1 through molecular docking, and explored the key role of TMP on the permeability of pulmonary capillary cells by detecting its related proteins. Muise AM (Muise et al., 2011) showed that carriage of the risk allele of rs10951982 results in increased Rac1 expression, and increased expression of Rac1 was associated with susceptibility to colitis. So there are human genetic polymorphisms in Rac1/LIMK1 that could predispose them to sepsis. Then we study the mechanism of LPS induced acute lung injury. Our findings suggest that TMP reduces pulmonary capillary hyperpermeability by inhibiting the Rac1/LIMK1 signaling pathway (Figure 8). Rac1 is thought to be a regulator of cytoskeletal remodeling (Schell and Huber, 2017; Li et al., 2018). A key enzyme in actin remodeling, LIMK1 belongs to the Ser/Thr family of serine/threonine (Ser/Thr) kinases. Activation of Rac1 phosphorylates and activates LIMK1, thereby regulating the actin cytoskeleton (Jia et al., 2016; Li and Wang, 2020; Duncan et al., 2021). This process suggests that the Rac1/LIMK1 signaling pathway affects endothelial permeability by regulating cytoskeletal remodeling (Ma et al., 2019; Zhang S. et al., 2020).

We observed higher serum levels of TNF-α, IL-6, and IL-1β in murine ALI, whereas the levels of the cytokines were downregulated after TMP administration. Additionally, MPO activity of the lungs in murine ALI was significantly decreased after TMP treatment. Because our focus was on endothelial permeability, we did not independently validate the signaling pathways involved in inflammation, but we present a solid foundation for future studies on TMP treatment.

To demonstrate that TMP inhibits both HUVEC skeletal remodeling and pulmonary capillary hyperpermeability, we designed different experiments to assess endothelial permeability. Firstly, immunofluorescence staining showed no increased and thickened F-actin fibers in normal cells. After LPS stimulation, F-actin fibers were increased and thickened. Compared to the LPS alone group, the corresponding reduction of F-actin in the low-dose TMP-pretreated group (15 μg/ml) was consistent with the trend in the positive control group that used dexamethasone. Similar results were seen from the TEER and permeability experiments. The low-dose TMP (15 μg/ml) and dexamethasone groups showed increased TEER and decreased permeability compared to the LPS group. Our previous research supports this conclusion, which showed that TMP can treat ALI by alleviating pathologic lung damage and reducing endothelial permeability, but the exact mechanism remains unclear. Because endothelial cell permeability is associated with cytoskeleton proteins, we further observed the Rac1 protein involved in the cytoskeleton and the change in LIMK1. We found that the effect of TMP on endothelial permeability was realized through inhibition of the Rac1/LIMK1 signaling pathway causing contraction and aggregation of F-actin. Our results show that TMP prevents LPS-induced tight junction impairment in C57 mice by inhibiting Rac1/LIMK1 pathway, which reverses cytoskeletal remodeling and prevents downregulation of junction proteins including ZO-1.

## Conclusion

TMP can reduce LPS-induced endothelial cell permeability and pulmonary inflammation. This inhibits pulmonary dysfunction in mice with ALI and HUVEC cytoskeletal remodeling. Cytoskeleton remodeling is inhibited *via* the Rac1/LIMK1 signaling pathway, further inhibiting vascular hyperpermeability. TMP can also inhibit tight junction damage in mice with ALI by enhancing tight junction protein expression. Acute lung injury is accompanied by inflammation and oxidative stress. Therefore, a further study is needed to confirm whether TMP inhibits oxidative stress in ALI.

## Data Availability

The original contributions presented in the study are included in the article/Supplementary Material, further inquiries can be directed to the corresponding author.
