# Supplementary material for: Tetramethylpyrazine ameliorates acute lung injury by regulating the Rac1/LIMK1 signaling pathway
Source: Front Pharmacol. 2023 Jan 6;13:1005014. doi: 10.3389/fphar.2022.1005014 (PMC9859661; doi:10.3389/fphar.2022.1005014)
Supplement: Supplementary file 4 [file Table1.DOCX]

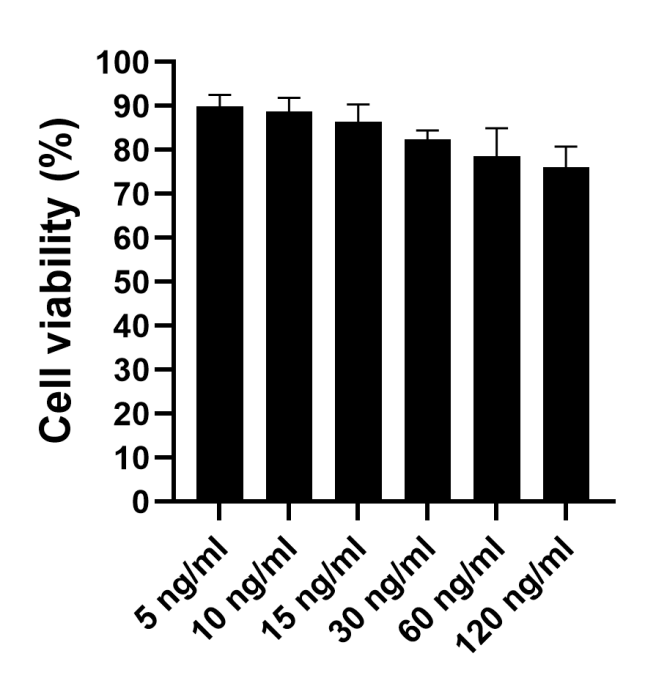


Figure S1: TMP of different concentrations (5 ng/ml-120ng/ml) after being incubated with HUVEC cells for 24 h determined by CCK-8 assay.


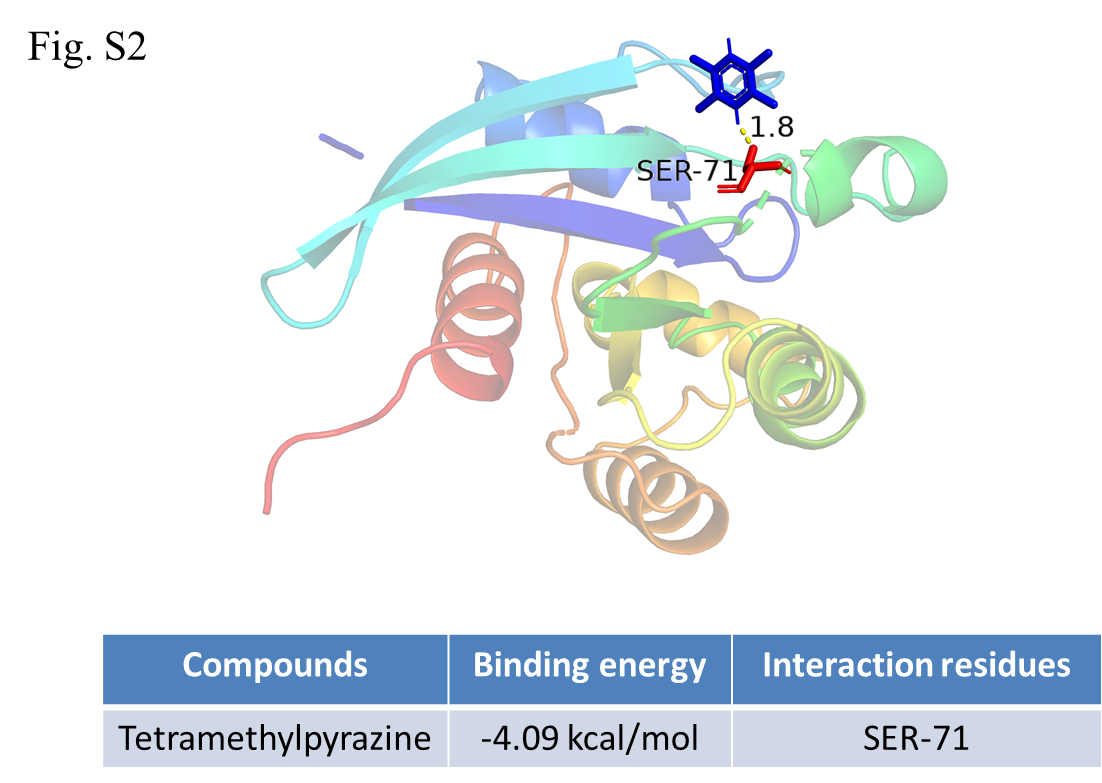


Figure S2: Molecular docking assay of Tetramethylpyrazine and Rac1. A potential binding pocket residues (SER-71) was predicted.
